# Muscle Strength and Power Improve Despite Small Increase in Quadriceps Cross-Sectional Area in Massive Knee Joint Destruction: A Two-Year Follow-Up Case Report

**DOI:** 10.3390/muscles5040075

**Published:** 2026-10-13

**Authors:** Carlos Cruz-Montecinos, Rodrigo Núñez-Cortés, Pablo Becerra-Yañez, Andrés Oyarzún, Joaquín Calatayud

**Affiliations:** 1 Department of Physical Therapy, Faculty of Medicine, University of Chile, Santiago 8380453, Chile; carloscruz@uchile.cl (C.C.-M.);; 2 Transversal Group on Rare, Uncommon, and Orphan Diseases (GERPOH-UChile), Faculty of Medicine, University of Chile, Santiago 8380453, Chile; 3 Kinesiology Unit, Hospital San José, Santiago 8380419, Chile; 4 Traumatology Unit, Hospital San José, Santiago 8380419, Chile; 5 Exercise Intervention for Health Research Group (EXINH-RG), Department of Physiotherapy, University of Valencia, 46010 València, Spain; joaquin.calatayud@uv.es

**Keywords:** haemophilic arthropathy, isometric exercise, resistance training, muscle strength, case report

## Abstract

Joint damage may result from different conditions, including osteoarthritis, rheumatoid arthritis, infection, and haemophilic arthropathy. Long-term effects of resistance training in individuals with massive joint destruction remain poorly documented. We report a case of a patient with haemophilia and massive joint destruction who completed a two-year home-based knee-extension programme. Training was performed twice weekly and included biofeedback-guided isometric knee extension at 80% of maximal voluntary isometric strength, followed by a closed-chain exercise using elastic resistance. Structural knee damage and quadriceps cross-sectional area (CSA) were assessed using computed tomography (CT). Pain, knee-extension strength, quadriceps CSA, and 30-s sit-to-stand performance were assessed at baseline and at two-year follow-up. Pain during active knee extension decreased from 6/10 to 2/10, and pain during daily activities decreased from 6/10 to 4/10. Knee-extension strength increased from 7 kgf to 20 kgf, representing a 186% increase. The 30-s sit-to-stand performance improved from 14 to 22 repetitions and estimated relative sit-to-stand muscle power increased from 3.4 to 5.3 W/kg, representing a 57% increase. Notably, quadriceps CSA increased by only 6% (from 1954.9 to 2078.6 mm^2^). No bleeding episodes or other adverse events were reported. After two years of strength training, marked gains in muscle strength and power were observed despite a small increase in quadriceps cross-sectional area. This case report highlights the need for future long-term follow-up studies of strength training in individuals with advanced joint damage.

## 1. Introduction

Severe joint damage may result from different conditions, including osteoarthritis, rheumatoid arthritis, infection, and haemophilic arthropathy [1,2]. Haemophilic arthropathy is particularly relevant because insufficient access to factor VIII or IX replacement therapy during childhood and adolescence can lead to recurrent intra-articular bleeding. Repeated exposure of the joint to blood promotes synovial inflammation, iron deposition, and progressive cartilage and bone damage [3,4], which in advanced cases may be more severe than that typically observed in degenerative or inflammatory joint diseases [5]. In these conditions of joint damage, resistance exercise is crucial to improve muscle function and pain [6,7,8]. However, the long-term effects of this treatment in individuals with massive joint destruction remain poorly documented, particularly regarding gains in muscle hypertrophy, strength and power. Nevertheless, research on muscle weakness has largely concentrated on atrophic changes, implicitly favouring structural over functional endpoints. This bias is problematic because strength and power are influenced not only by muscle size but also by neural factors (e.g., motor unit discharge rate) [9,10]. Demonstrating that strength and power can improve despite limited muscle hypertrophy would have important clinical implications, as functional gains may still be achievable in patients with advanced joint damage in whom substantial muscle hypertrophy may be difficult to attain.

Therefore, the aim of this case report was to describe the changes in knee extensor strength, lower-limb muscle power, pain, and quadriceps cross-sectional area over a two-year follow-up following a home-based resistance exercise programme in a patient with severe haemophilia A and massive knee joint destruction.

## 2. Methods

### 2.1. Patient Information

**Figure 1:**
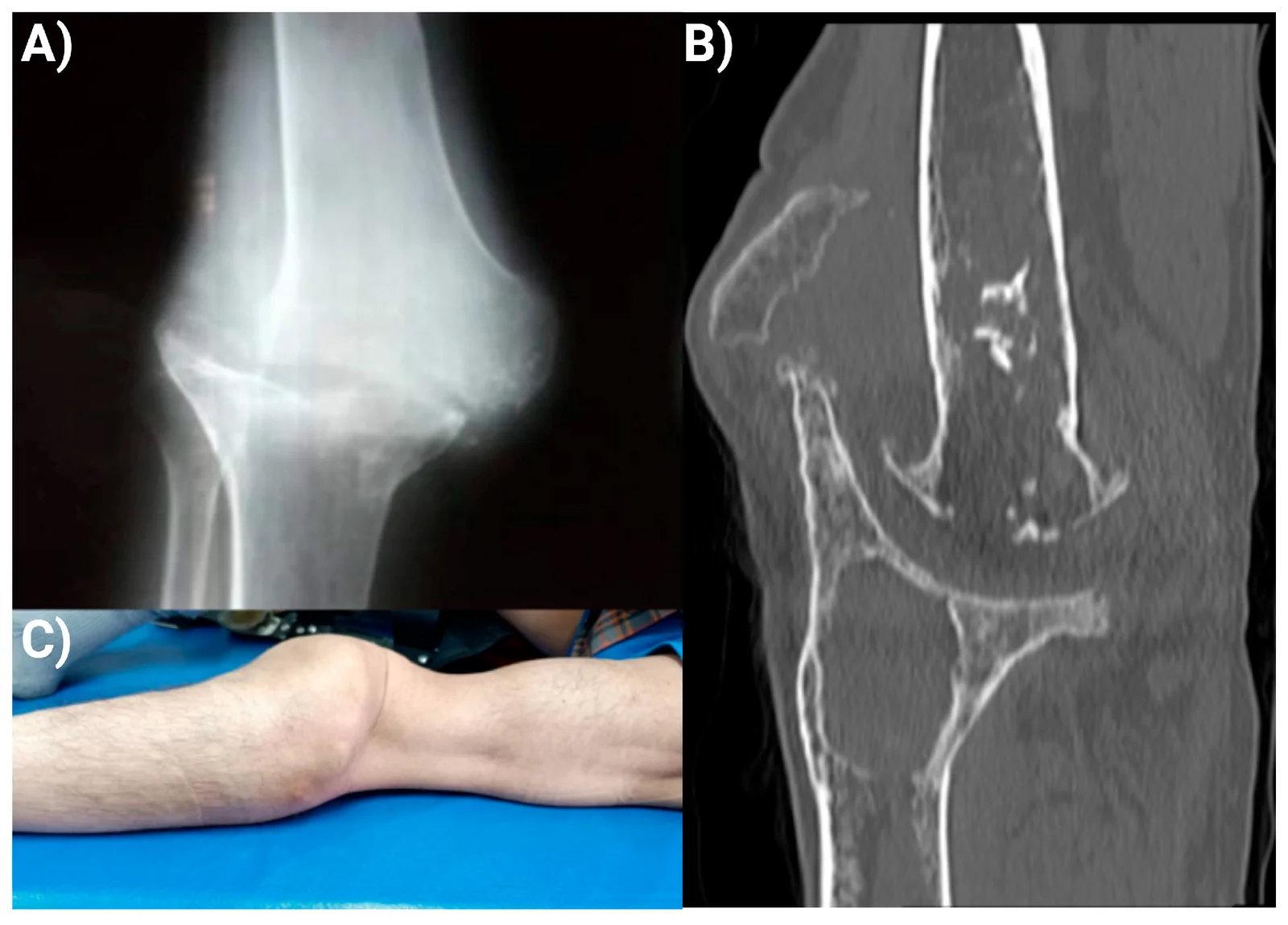
Baseline clinical and imaging assessment of the affected knee. (**A**) Anteroposterior radiograph demonstrating severe degenerative changes and advanced joint destruction. (**B**) Coronal computed tomography image confirming extensive structural damage involving the distal femur and proximal tibia. (**C**) Clinical photograph showing marked muscle atrophy of the affected knee.

This case report describes a 53-year-old man with severe haemophilia A (factor VIII activity < 1% according to the medical record) and a 20-year history of advanced left-knee arthropathy. He had undergone contralateral total knee arthroplasty in 2008. He received prophylactic factor VIII replacement therapy (2000 IU three times per week) and used Celebra^®^ (celecoxib) as needed for pain management. Analgesic use immediately before or after exercise sessions was not systematically recorded. The patient was 168 cm tall and weighed 54 kg. He presented with pain during active knee extension (6/10 on a numerical rating scale [NRS]), during daily activities (6/10 NRS), and marked knee extensor weakness. At the initial assessment, the treating orthopaedic surgeon was evaluating definitive surgical management, including total knee arthroplasty with a tumour-type prosthesis or knee arthrodesis. The treating orthopaedic surgeon reviewed the clinical and imaging findings (Figure 1). The lesion was described as Peking Union Medical College Hospital (PUMCH) type III (mixed soft-tissue and bone involvement) [11] and as an Anderson Orthopaedic Research Institute (AORI) type III bone defect, reflecting major metaphyseal bone loss [12]. These systems were used descriptively because they were developed for haemophilic pseudotumours and revision knee arthroplasty bone defects, respectively.

He walked with a stabilising knee brace and a cane and showed passive knee range of motion from −10° extension to 110° flexion. The patient was self-employed as a furniture maker and worked from home. He was married with two children and remained independent in activities of daily living. He had no prior experience with comparable biofeedback-guided strength training. This case report was prepared in accordance with the CARE guidelines [13].

### 2.2. Outcomes

Motor function was assessed using knee-extension force and 30-s sit-to-stand test performance. Maximal voluntary isometric contraction (MVIC) force during knee extension was assessed using a portable Bluetooth dynamometer (Progressor 200, Tindeq AS, Leknes, Norway) [14] and reported as absolute force (kgf) and relative force normalized to body mass (kgf/kg). The dynamometer was secured to the distal leg using a Velcro strap, positioned at a standardised distance proximal to the lateral malleolus, and aligned perpendicular to the tibia. For strength assessment, the patient performed maximal voluntary isometric contractions, and the highest recorded force was retained for analysis. At baseline, maximal isometric knee-extension strength in the affected limb was 7.0 kgf (0.13 kgf/kg body mass). Because contralateral-limb strength was measured only at the end of the two-year follow-up (28.6 kgf), a longitudinal assessment of interlimb asymmetry was not possible. For descriptive purposes, the strength difference relative to this single contralateral reference was calculated as [(contralateral − affected)/contralateral] × 100.

Lower-limb functional performance was assessed using the 30-s sit-to-stand (30s-STS) test with a standardised 0.43-m chair. The patient was instructed to rise from and return to a seated position as many times as possible within 30 s, and the total number of completed repetitions was recorded. Relative sit-to-stand muscle power (W/kg) was estimated from body mass, body height, chair height, and the number of repetitions completed during the test, using the previously described equation [15], which has also been applied in people with haemophilia [16].

**Figure 2:**
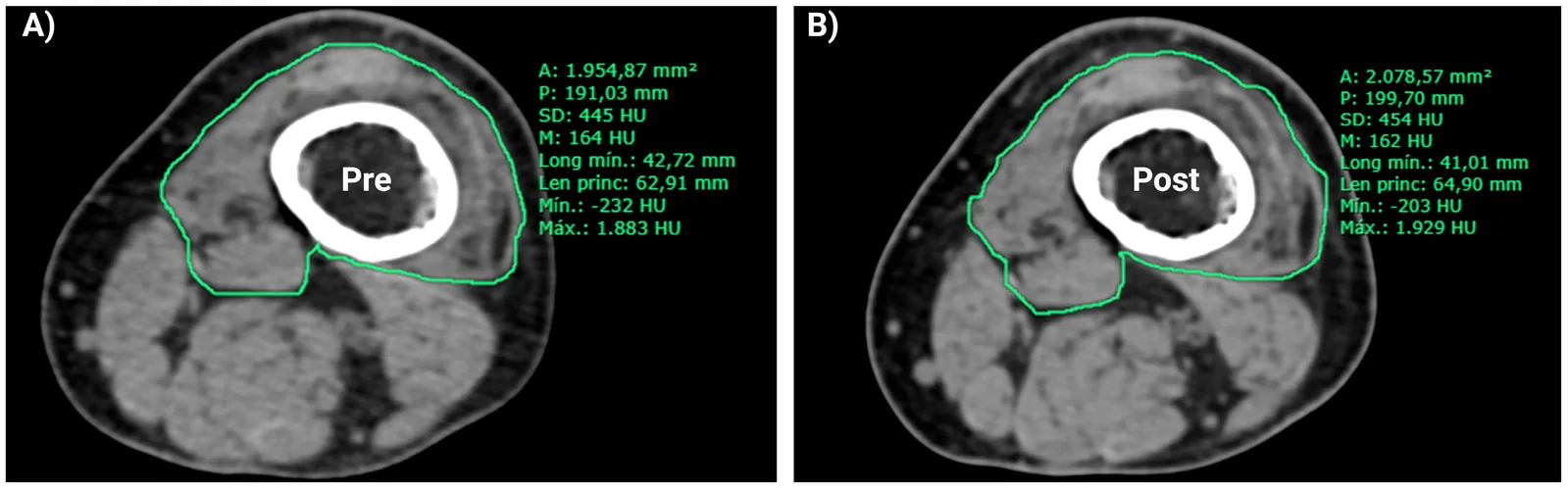
Pre–post changes in quadriceps muscle cross-sectional area. Representative axial computed tomography images obtained 100 mm proximal to the knee joint line: (**A**) before (Pre) the intervention; (**B**) after (Post) the intervention. The green contour delineates the quadriceps muscle used for cross-sectional area analysis.

Quadriceps cross-sectional area (CSA) was quantified from one axial CT slice located 100 mm proximal to the knee joint line; the same anatomical level was used at baseline and follow-up (Figure 2 and Figure S1). Because CT imaging did not cover the entire thigh, whole-muscle volume could not be assessed, and contralateral quadriceps CSA was unavailable.

### 2.3. Therapeutic Intervention

Prophylactic factor treatment was administered on training days as part of bleeding-risk management, and celecoxib use and orthopaedic follow-up continued as part of concurrent medical care. The intervention consisted of home-based isometric knee-extension training guided by real-time visual force biofeedback using a portable Bluetooth dynamometer (Progressor 200, Tindeq AS, Leknes, Norway), followed by a closed-chain exercise performed with elastic resistance. Face-to-face follow-up sessions were conducted approximately once per month to monitor exercise technique, progression, pain tolerance, adherence, and medical status. The isometric training protocol was delivered through the manufacturer’s Tindeq Progressor smartphone application, which was used to set the exercise protocol, provide real-time visual biofeedback, and automatically record and store all training sessions. Training was performed in a seated position with the hip and knee flexed to 90°. The initial structured phase lasted 8 weeks and was performed twice weekly. Each session included 4 sets of 10 repetitions, with a 3-s isometric hold per repetition and 2 min of rest between sets. The target intensity was set at 80% of MVIC. MVIC was assessed at the beginning of each session and used to establish the individual target force for visual biofeedback, expressed in kilogram-force (kgf). The progression of biofeedback was focused on improving the speed of force generation, reducing the time needed to reach the target force from approximately 3–5 s to 1 s. After the biofeedback-guided isometric training, the patient performed a closed-chain dynamic quadriceps exercise in standing using an elastic band. This exercise was selected because the patient was unable to tolerate loaded open-chain knee-extension exercises. The closed-chain exercise consisted of 3 sets, separated by 2-min rest intervals. During the initial sessions, the patient completed approximately 12–15 repetitions per set at a perceived exertion of 4–5 on the Borg CR-10 scale. Resistance was progressively increased so that approximately 8–12 repetitions elicited a perceived exertion of 7–8, while maintaining 2–3 repetitions in reserve at the end of each set. After the initial 8 weeks, both exercises (i.e., biofeedback-guided isometric and closed-chain exercises) were continued twice weekly for the remainder of the two-year follow-up period using the same progression criteria. Training loads for both exercises were reviewed and adjusted approximately once per month during face-to-face follow-up sessions at the hospital to ensure progressive loading. The load was always adjusted to maintain tolerable exercise pain (approximately 4–5/10 NRS). The patient tolerated the intervention without reporting increased pain, bleeding episodes, or other adverse events.

### 2.4. Ethics Approval and Informed Consent

The exercise programme was delivered as part of routine clinical care. The clinical data were collected retrospectively. After obtaining the patient’s written informed consent, the case was submitted to the Ethics Committee of the Metropolitan Health Service, Santiago, and ethical approval was granted on 24 June 2026. The patient provided written informed consent for publication of the clinical information and accompanying images.

## 3. Results

### 3.1. Clinical Findings and Follow-Up

**Figure 3:**
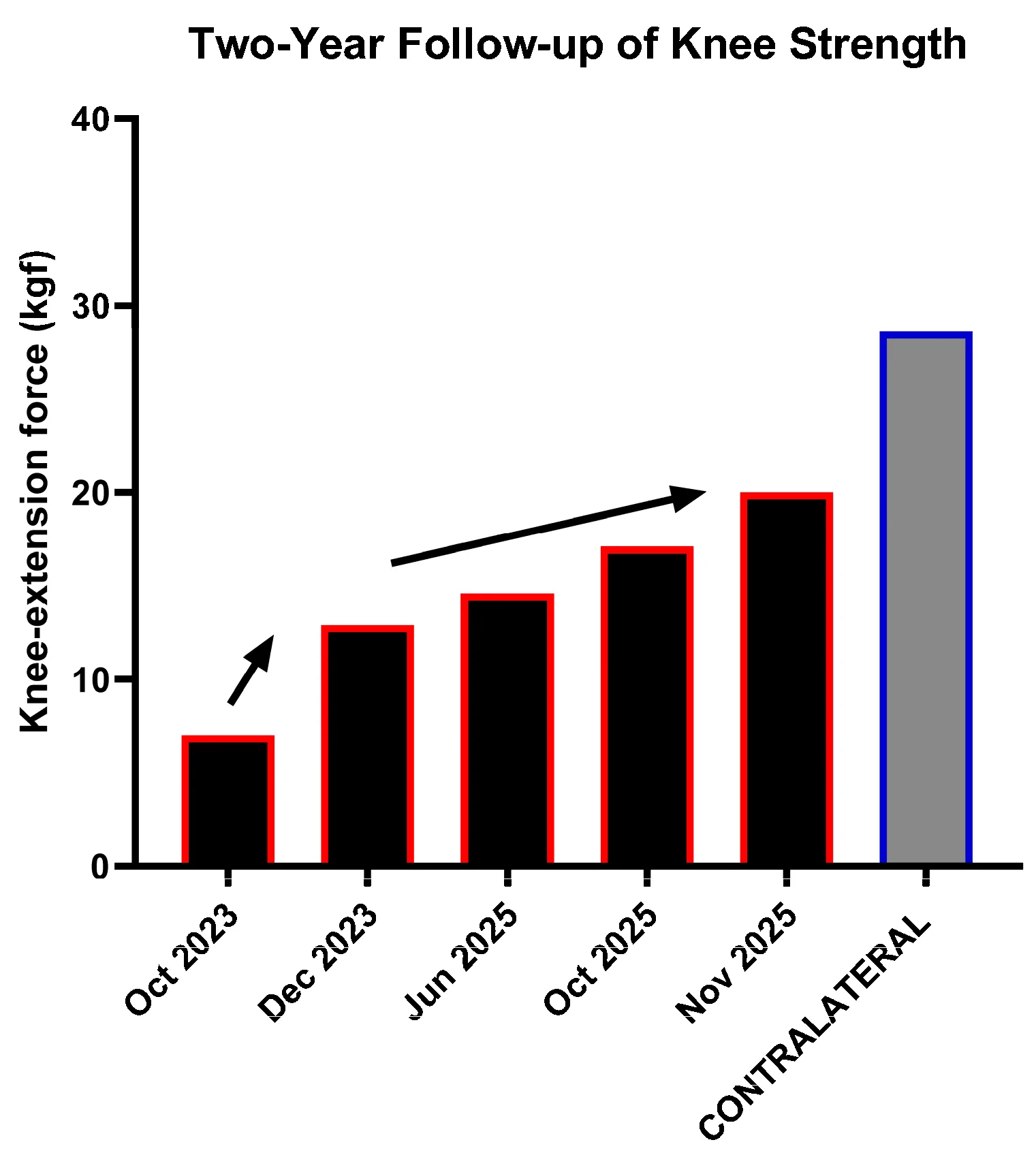
Two-year follow-up of knee extensor strength. Knee extensor strength progressively increased throughout the intervention period and remained below the contralateral limb at the final follow-up. Black bars with red outlines represent the treated limb, and arrows indicate the increase in knee extensor strength over time. The grey bar represents the contralateral limb strength measured at the end of the two-year follow-up.

**Figure 4:**
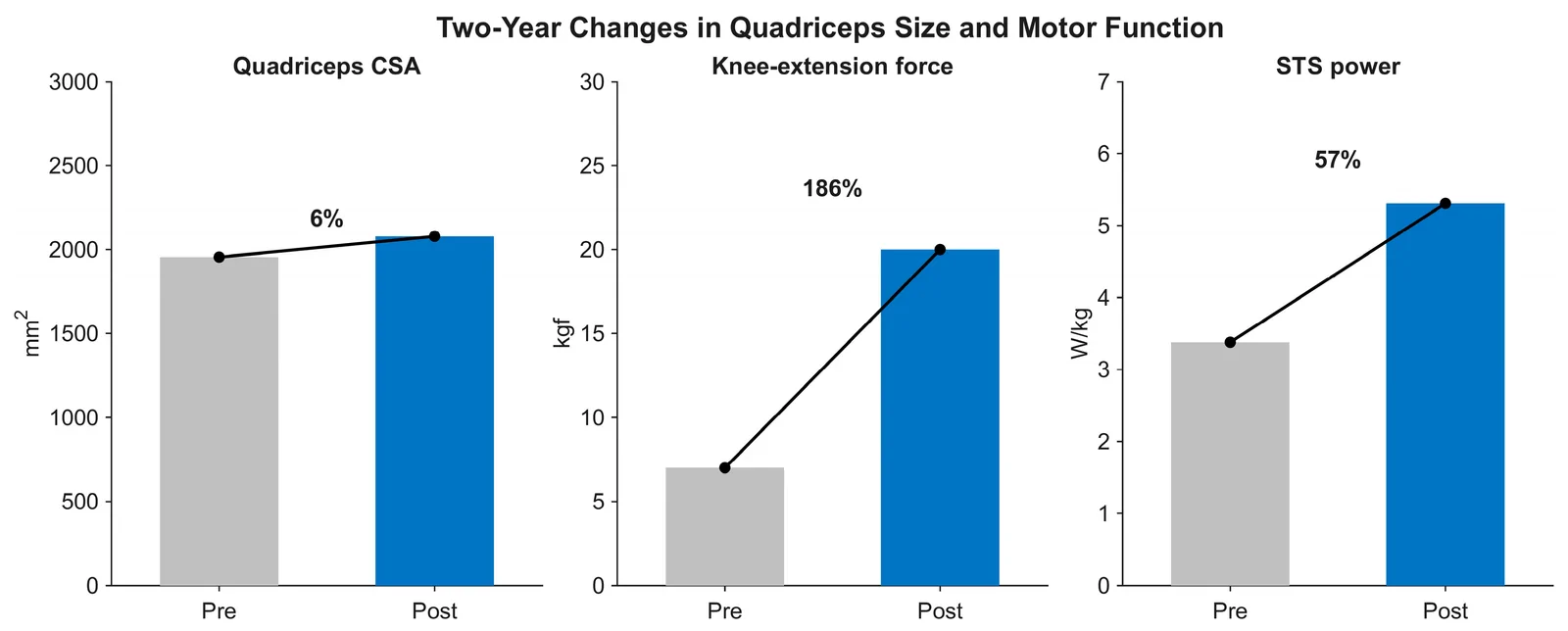
Pre–post changes in quadriceps muscle size, knee-extensor strength, and functional power. Quadriceps cross-sectional area (CSA) increased by 6%, whereas knee-extensor force and sit-to-stand (STS) power increased by 186% and 57%, respectively.

Overall adherence to the prescribed training sessions was about 85% over the two-year intervention. Using the contralateral strength measured at follow-up as a single reference, the estimated strength difference was 76% at baseline and 30% at follow-up (Figure 3). Absolute changes in pain, knee-extension strength, STS power, and quadriceps CSA are shown in Table 1 and Figure 4. Knee-extension strength increased by 186%, STS power by 57%, and quadriceps CSA by 6%. No adverse events were reported during the strength-training programme.

### 3.2. Patient Perspective

The patient reported feeling safer and more confident with the intervention. He described that the programme helped him better understand his capacity to generate force with the affected leg and to exercise with less pain. Over the two-year follow-up, being able to visualise his progress through the mobile biofeedback system, together with regular support from the physiotherapist, gave him confidence to continue improving his routine and use the leg in daily life activities. Being able to visualise his force levels every session and progress through the mobile biofeedback system encouraged him to challenge himself and continually try to improve his performance. He also reported a reduction in persistent knee pain and those episodes of worsening knee symptoms became less frequent than before the programme. He is currently awaiting the medical team’s decision regarding the most appropriate surgical option (e.g., total knee arthroplasty with a tumour-type prosthesis or arthrodesis) but feels confident that the strength gains achieved represent meaningful progress and may help prepare him for postoperative recovery.

## 4. Discussion

Reports of strength training in individuals with massive joint destruction are scarce in the literature. After two years of a home-based knee-extension programme, this case showed substantial and sustained improvements in knee-extensor strength and functional muscle power in a patient with massive knee joint destruction, despite a small increase in quadriceps CSA. This intervention was completed without reported bleeding episodes or other adverse events in this patient. These findings provide new insight into how resistance training can be implemented despite severe structural joint damage, with potential to improve muscle function and motor performance.

### 4.1. Changes in Quadriceps Muscle Size

The main finding of this case report is the marked improvement in strength and power despite only a modest increase in quadriceps CSA. However, the programme was designed primarily to improve force production rather than to maximise hypertrophy; therefore, a modest morphological response was plausible. Although muscle hypertrophy may contribute to strength gains, the magnitude of improvement observed here is unlikely to be explained by morphological change alone [17]. In healthy individuals, systematic reviews indicate that resistance training induces approximately 5–17% increases in quadriceps muscle size (e.g., cross-sectional area and muscle thickness) [18,19]. The 6% increase in quadriceps cross-sectional area observed in the present case was similar in magnitude to the 7–8% increases reported after 12 weeks of supervised resistance training in women with knee osteoarthritis [20]. Recent evidence suggests that resistance-training-induced hypertrophy follows a nonlinear trajectory (i.e., rapid early gains followed by progressively smaller incremental gains), with estimated increases in muscle size of approximately 7.5% at 12 weeks, 10.5% at 36 weeks, and 12.6% at 78 weeks [21]. Long-term hypertrophic adaptations in individuals with advanced joint destruction remain unclear, warranting further investigation in this population.

### 4.2. Gains in Strength and Power

The 186% increase in knee-extension strength observed in this case exceeds the magnitude commonly reported in joint disease. Previous studies in people with haemophilia have reported strength gains of approximately 10% to 30% after exercise programmes using isometric training, elastic bands, or conventional resistance exercises [7]. In people with osteoarthritis, strength gains greater than 30% have been considered clinically meaningful [8]. Similarly, the 30-s sit-to-stand performance improved by 8 repetitions, exceeding the commonly used threshold of 2 repetitions for clinically meaningful change [22]. Estimated relative sit-to-stand muscle power increased by 57%, which is also notable, given that power-training studies in older adults commonly report improvements of approximately 10% to 40% [23,24,25], and changes around 12% have been considered clinically relevant [24]. Evidence regarding expected changes in muscle power in people with advanced joint destruction remains limited, making this finding particularly relevant. The prolonged duration of the intervention may partly explain the marked gains observed in this case. Most studies in people with joint damage assess resistance training effects after only 8–12 weeks, which may not be directly comparable with two years of sustained training. Other factors, such as reduced pain-related inhibition, task familiarisation, and the very low baseline strength, may also have contributed to the functional gains observed. Long-term consistency may therefore have been an important contributor to the improvements in strength [26].

### 4.3. Functional and Clinical Implications

To explain the discrepancy between the percentage gains in quadriceps muscle size and motor function (i.e., knee strength and STS power), non-morphological adaptations should also be considered. The marked improvement in muscle strength despite only modest quadriceps hypertrophy is consistent with the concept that voluntary strength gains result from both neural adaptations and muscle hypertrophy [27]. Neural adaptations may include increased neural drive to the muscle, enhanced motor unit recruitment, and higher motor unit discharge rates (i.e., rate coding) [9,10,28]. Reduced pain-related muscle inhibition and fear of movement may also have contributed to the observed gains in muscle strength and power [16,29]. These mechanisms may be particularly relevant in individuals with severe joint damage, in whom structural compromise, pain, and fear regarding adverse events can limit voluntary activation and exercise participation. In this context, resistance training may improve force production, joint stability, motor control, and confidence in movement and may also be accompanied by changes in inflammatory pathways [6,30].

This case has three main clinical implications. First, modest increases in quadriceps muscle size may still be achieved despite massive structural joint damage. However, this finding should be interpreted in the context of the two-year follow-up, as the long duration of training may have contributed to the observed morphological adaptation. Second, gains in muscle mass may not occur in parallel with improvements in strength and power. Although muscle size remains an important component of muscle function, the marked increases in strength and power observed in this case despite only a modest increase in quadriceps cross-sectional area highlight the need to routinely monitor muscle strength and power, rather than relying solely on measures of muscle morphology. Third, home-based resistance training with biofeedback may represent a feasible therapeutic approach for supporting long-term adherence and maintaining regular training exposure. Further longitudinal studies are needed to evaluate this type of intervention in larger samples and across different populations with chronic or severe joint damage.

### 4.4. Strengths and Limitations

This case report includes the two-year follow-up of a strength-training intervention in a rare case of massive knee joint destruction, the use of an innovative home-based, biofeedback-guided training strategy, and the assessment of both functional and morphological outcomes. However, several limitations should be acknowledged. First, as a single case report, causality cannot be established, and the findings should be interpreted as hypothesis-generating for future long-term studies comparing quadriceps muscle size, strength, and power in patients with severe joint damage. Similarly, the absence of reported adverse events cannot establish safety or be generalised to other patients with severe joint damage. Second, neural activation and quality of life were not assessed, limiting the interpretation of the mechanisms underlying the observed improvements and whether these changes translated into better daily functioning or quality of life. Third, the very low baseline strength, task familiarisation, celecoxib use, and possible unmeasured changes in physical activity should also be considered as possible explanations for these results. Fourth, the contralateral limb was not longitudinally monitored because it was not the primary clinical problem. Contralateral strength was measured only at the end of the two-year follow-up, and contralateral quadriceps CSA was not assessed longitudinally. The reported strength differences should therefore be interpreted as descriptive estimates based on a single contralateral reference value, limiting within-patient comparison and the interpretation of the observed changes. Finally, morphological changes were assessed only using CSA; additional measures, such as muscle volume, intramuscular fat content, or muscle architecture, may provide further insight [31,32].

## 5. Conclusions

Reports of strength training in individuals with massive joint destruction are scarce in the literature. This case report emphasises that, in the context of massive joint destruction, two years of resistance training may be associated with marked gains in muscle function (strength and power) despite a small increase in quadriceps cross-sectional area. This case report highlights the need for future long-term follow-up studies of strength training in individuals with advanced joint damage.

## Figures and Tables

**Table 1:** Follow-up and Outcomes.

Outcome	Baseline	Two-Year Follow-Up	Change
Pain during active extension (NRS)	6/10	2/10	−4 points
Pain during daily activities (NRS)	6/10	4/10	−2 points
Knee-extension force	7 kgf	20 kgf	+186%
Relative knee-extension force	0.13 kgf/kg	0.37 kgf/kg	+186%
Estimated strength difference relative to the contralateral limb	76%	30.0%	Improved
30-s sit-to-stand	14 repetitions	22 repetitions	+57%
30-s sit-to-stand power	3.4 (W/kg)	5.3 (W/kg)	+57%
Quadriceps CSA	1954.9 mm^2^	2078.6 mm^2^	+6%
Adverse events during the intervention	—	None reported	—

Note: Strength difference was estimated using contralateral strength measured only at follow-up.

## Data Availability

The data supporting the findings of this case report are available from the corresponding author upon reasonable request.

## Supplementary Materials

The following supporting information can be downloaded at: https://www.mdpi.com/article/10.3390/muscles5040075/s1, Figure S1: Computed tomography scout view showing the standardized quadriceps cross-sectional area measurement level, 100 mm proximal to the knee joint line.
