# Supplementary material for: Muscle Strength and Power Improve Despite Small Increase in Quadriceps Cross-Sectional Area in Massive Knee Joint Destruction: A Two-Year Follow-Up Case Report
Source: Muscles. 2026 Oct 13;5(4):75. doi: 10.3390/muscles5040075 (PMC13692431; doi:10.3390/muscles5040075)
Supplement: Supplementary file 1 [file muscles-05-00075-s001.zip › muscles-4473953-supplementary.pdf]

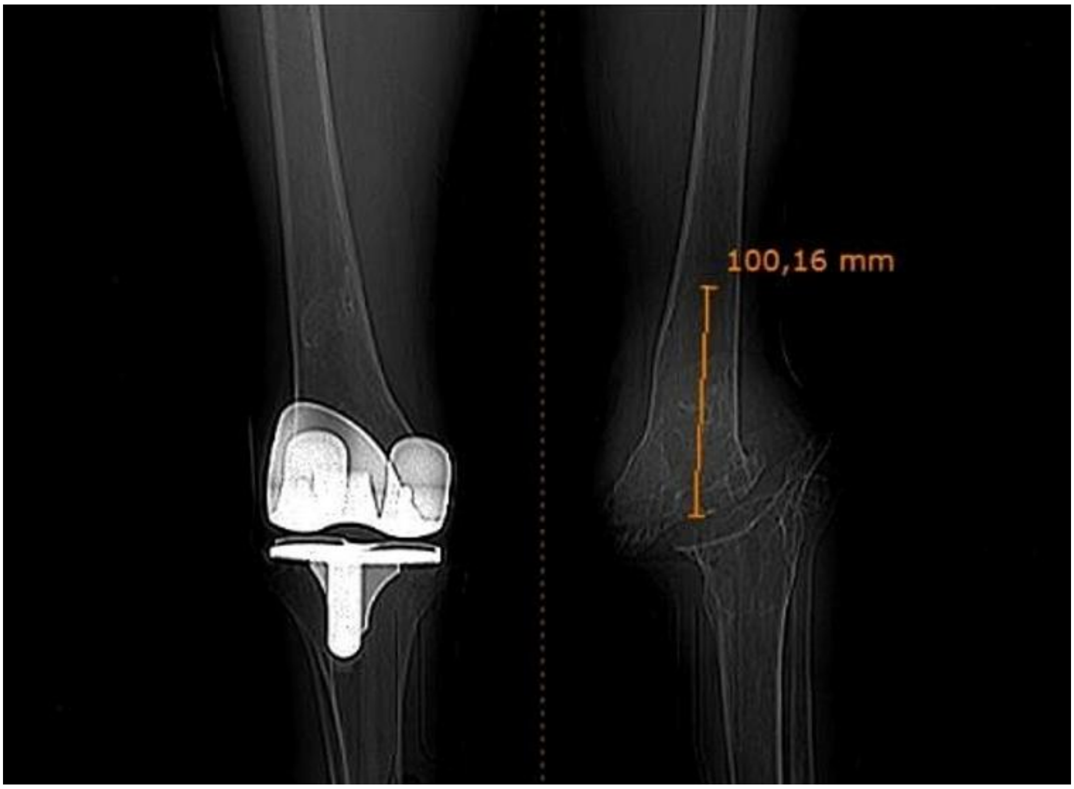

Figure S1. Computed tomography scout view showing the standardized quadriceps cross-sectional area measurement level, 100 mm proximal to the knee joint line.
